# Fractal Plasmons on Cantor Set Thin Film

**DOI:** 10.3390/e21121176

**Published:** 2019-11-29

**Authors:** David Ziemkiewicz, Karol Karpiński, Sylwia Zielińska-Raczyńska

**Affiliations:** Institute of Mathematics and Physics, UTP University of Science and Technology, Al. Prof. S. Kaliskiego 7, 85-789 Bydgoszcz, Poland; karol.karpinski@utp.edu.pl (K.K.); sziel@utp.edu.pl (S.Z.-R.)

**Keywords:** surface plasmons, fractals, entropy

## Abstract

The propagation of surface plasmon–polaritons is investigated in a metallic, fractal-like structure based on Cantor set. The dynamic of plasmonic modes generating on the Cantor structure is discussed in the context of the setup geometry. The numerically obtained reflection spectra are analyzed with the box-counting method to obtain their dimension, which is shown to be dependent on the geometry of the plasmonic structure. The entropy of the structure is also calculated and shown to be proportional to the dimension. Presented analysis allows for extracting information about fractal plasmonic structure from the reflectance spectrum. Predictions regarding the experimental observation of discussed effects are presented.

## 1. Introduction

Over the past decades, the study of electromagnetic waves propagating at the interface between a metal and dielectric has been of significant interest. In such a physical system the energy of the electromagnetic field is confined to sub-wavelength size supporting localized surface plasmon polariton (SPP) modes. Their unusual properties depend on the metal and geometry of the material structure, which in fact plays a crucial role in local electromagnetic enhancement and tuning the resonance positions. Such interdependences have been recently studied regarding the impact of the nonlocality of the surface structure on the dispersion relation of SPP [1]. A lot of work has been devoted to the examination of plasmonic properties of various metal surfaces with specific geometrical shapes, e.g., periodic V-grooves [2], slabs with periodic grating [3] or to investigate propagation of a confined field in periodically corrugated waveguides [4]. Carefully designed metasurfaces offer new opportunities for tunable unidirectional excitation of SPPs whose propagating direction depends on the helicity of incident light [5]. The recent advancements in the research of geometric metasurfaces and their applications in ultrathin optical devices have been presented in [6]. The idea of examining fractal geometries was a natural step forward in plasmonic studies. In recent years there has been continuous interest in the development of fractal metamaterials, which could be used in high-gain, compact, multiband antennas [7,8,9].

Significant research effort has been devoted to fractal space-time systems, starting from analysis of diffusion in fractal space [10,11]. Interesting results can be obtained even in simple wave propagation problems; Berry has proved that the diffraction patterns caused by fractal objects are fractals themselves [12], which has been since experimentally confirmed [13]. Cherny et al. [14] studied the small-angle scattering in generalized Cantor set fractals.

There is an inherent connection between entropy and fractal dimension [15]; recently, Chen [16] has used this connection for the study of urban systems, which are natural, self-organizing, fractal structure. While there are some studies of entropy of electromagnetic waves reflected from metallic structure [17], it seems that this paper is the first attempt to link the relevance of entropy of fractal systems where surface plasmons are excited.

It is important to note that realistic, fractal-like physical systems cannot contain infinitely small parts; if the construction is iterative, only a finite number of iterations is used. Examples of such structure are triadic-Cantor photonic crystals [18] and plasmonic superlattices [19] which can be analyzed using standard transfer matrix approach. Similarly, the finite number of Cantor set iterations in plasmonic structure allows one to approach the problem with classical optics and to use the standard form of Fourier transform.

These observations and studies inspire us to study plasmon propagation in fractal structures. Such systems have geometry which scales in a nontrivial way and is characterized by a non-integer dimension. One of the simplest examples of a fractal is the Cantor set [20]. The aim of our paper is to investigate the propagation of plasmons generated by an electromagnetic wave incident at the quasi-periodic Cantor-like metallic layer. In our analysis, the structure is generated in finite, but sufficient number of iterations so that the finest structures are much smaller than the wavelength. This is particularly important for plasmonic structure, as it is well known that SPPs can interact with sub-wavelength surface features. Therefore, one may expect a very rich system dynamics in the case of fractal metallic structures. By using the Cantor set as a geometrically simple example of fractal structure, we use the well-established principles of SPP excitation in optical dipole antennas [21] and thin layers [22] to predict the number and frequency of SPP modes excited in the system. Then, we demonstrate that the reflection spectrum of fractal plasmonic structure can be interpreted as a fractal and characterized by a non-integer dimension, providing a new, useful measure in spectroscopy of plasmonic systems. Our work may unlock a novel opportunity to elicit information about the surface structure from the reflection spectrum of SPPs and therefore establishes a link between optical properties and different fractal geometries. The results are general and applicable to a wide class of natural and fabricated systems characterized by a non-integer dimension. Although our considerations are purely theoretical, nevertheless the numerical simulations are based on the realistic model of the fractal-like structure of the silver film on the glass substrate; we believe that presented results can be an inspiration for the experimental investigations.

## 2. Theory

**Figure 1:**
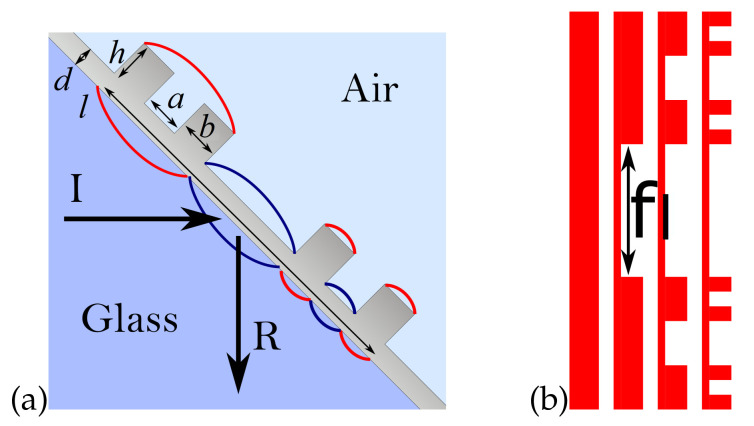
(**a**) Schematics of the considered plasmonic system, with propogation direction of incident (I) and reflected (R) waves. (**b**) First three iterations of Cantor set construction with structure length *l* and removed part fraction f=1/3.

Our setup consists of a metal layer with indentations, deposited on a glass substrate (Figure 1a). The minimum and maximum layer thickness are *d* and d+h respectively. The indentations are cut according to the iterative process of generalized Cantor set construction [20] (Figure 1); at every iteration, a middle part of the unmodified metal surface is cut, forming two thicker areas (“islands” with length *a*) and a middle, thinner area (“groove”, with length *b*). The size of the removed part is given by a fraction 0<f<1. Assuming that the total length of the structure is *l*, the length of grooves and islands obtained in the *i*-th iteration is respectively

(1) $$\begin{array}{ccc} a_{i} & = & l{\left[(1-f)/2\right]}^{i},\\ b_{i} & = & lf{\left[(1-f)/2\right]}^{i-1}. \end{array}$$

In our manuscript, we analyze the case in which the number of iterations is i=3 and f∈(0,0.4), where f=1/3 is the standard Cantor set. For f>0.4 and i>3, the size of the metallic parts quickly decreases, approaching the size of the unit cell in numerical simulation.

**Figure 2:**
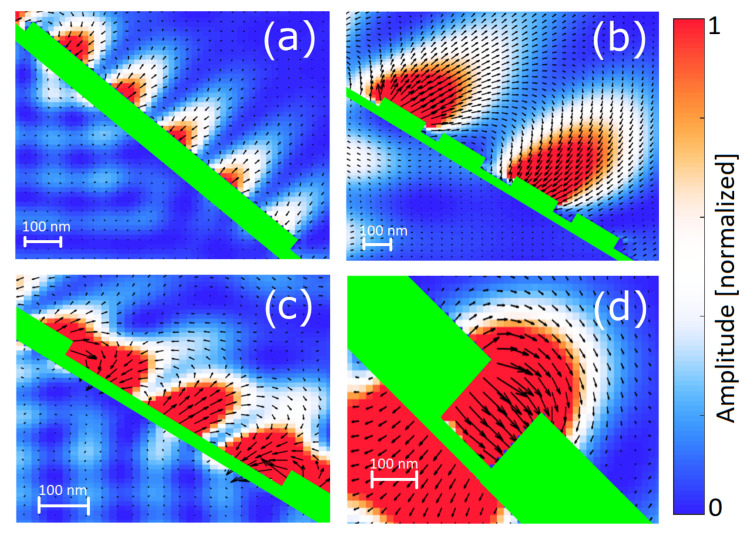
Various plasmonic modes forming on the Cantor structure. Color and arrows mark the electric field intensity $E^{2}$ and direction, respectively. The metallic structure is shown in green. (**a**) Island mode with b=2.5λ. (**b**) Low-frequency island mode spanning whole first iteration structure, ignoring the second and third iteration grooves. (**c**) Groove mode consisting of standing wave with a≈1.5λ and two additional intensity peaks on the island edges. (**d**) Narrow groove mode.

A TM-polarized incident wave (*I* on the Figure 1a) illuminates the surface from the glass side, at an angle $\theta =45^{\circ }$, in the Kretschmann configuration [23]. For some selected frequencies, the incident wave can excite multiple surface plasmon modes (marked by red and blue lines). One can expect that the strongest plasmonic resonances will have the form of standing waves, with multiples of half-wavelength matching the length of various horizontal surfaces $b_{i}$ in the structure, e.g.,

(2) $$N\lambda /2=b_{i},\;N\in N,\;i=0\ldots n,$$

where *i* is the iteration number, so that the distances range from the total structure length $b_{0}=l$ down to the smallest features $b_{n}$. Figure 2 shows the typical plasmonic modes obtained in a finite-difference time-domain (FDTD) simulation.

In Figure 2a one can see an example of “island” mode in the form of a standing wave, with wavelength being a fraction of the island length *b*. Typically for SPP, the electric field is perpendicular to the metal surface, drops exponentially with the distance from the metal and vanishes inside the metal. Interestingly, long-wavelength island modes can form over the grooves (Figure 2b). This means that in a Cantor structure created in *i* iterations, there will be SPP resonances corresponding to the structures obtained in iteration $i^{\prime }\leq i$ and the whole reflection spectrum is a sum over all iterations. Figure 2c depicts a typical “groove” mode. A standing wave is formed inside the groove. In addition, there are two intensity peaks forming on the island edges enclosing the groove. Due to this phenomenon, the efficient wavelength of the model is slightly longer than the allowed space inside the groove could permit. This effect becomes more pronounced as the groove size *a* decreases (Figure 2d); in such a case, the plasmonic mode changes the field configuration, with the electric field direction parallel to the metal-glass interface. Moreover, the wavelength is no longer dependent on the groove length and instead becomes proportional to the groove depth of *h*. One can also see that this type of mode is relatively weak—the electric field amplitude is comparable to the free-propagating wave on the other side of the metal surface. In contrast, the other depicted SPP modes are much stronger than the incident and reflected field. This is caused by the fact that for those modes, the electric field on the vertical walls (edges) of the structure is negligible, reducing power losses due to induced currents flowing along these walls and thus creating strong, standing wave patterns. The excitation of plasmons takes energy from the incident wave, reducing the amplitude of the reflected wave *R*.

For quantitative results, we recall the model presented in [24], where the incident field excites SPPs on a silver layer. The permittivity of silver is calculated with the Drude model

(3) $$\epsilon_{m}\left(\omega \right)=1-\frac{\omega_{p}^{2}}{\omega^{2}+i\gamma \omega },$$

with the plasma frequency $\omega_{p}=1930.5$ THz and damping ratio γ=31.35 THz obtained from fitting to the value $\epsilon_{m}\left(\lambda =589\;nm\right)=-13.3+0.883i$ [25]. The metal film is deposited on a glass substrate with $\epsilon_{g}=2.25$. To obtain the frequency of SPP modes, one has to consider the boundary conditions on the glass-metal and metal-air interfaces [22,26].

(4) $$\begin{array}{c} \frac{\kappa_{m}\tanh\left(\kappa_{m}\frac{d^{\prime }}{2}\right)}{\epsilon_{m}}=\frac{-\kappa_{a}}{\epsilon_{a}}=\frac{-\kappa_{g}}{\epsilon_{g}},\\ \frac{\kappa_{m}\coth\left(\kappa_{m}\frac{d^{\prime }}{2}\right)}{\epsilon_{m}}=\frac{-\kappa_{a}}{\epsilon_{a}}=\frac{-\kappa_{g}}{\epsilon_{g}}, \end{array}$$

where $\kappa_{j}=\sqrt{k^{2}-\epsilon_{j}\frac{\omega^{2}}{c^{2}}}$ denotes the component of the wave vector *k* parallel to the surface of the *j*-th medium with j∈{a,m,g} corresponding respectively to the air, metal and glass; $\epsilon_{j}$ are the relative electric permittivities of these substances and $d^{\prime }$ is the layer thickness ($d^{\prime }=d$ and $d^{\prime }=d+h$ for groove and island modes, respectively). The two above equations describe the so-called short-range and long-range SPPs. In this paper, we consider only the stronger, long-range modes. Furthermore, in the case of the Cantor set structure shown on the Figure 1a, there are two different types of modes corresponding to the layer thickness *d* and d+h respectively. By solving the Equation (4) numerically, we obtain a dispersion relation in a form ω(λ). This allows us to calculate the frequency of the modes with wavelengths given by (2), corresponding to the various distances in the structure given by Equation (1). Due to this close correspondence between the structure and the surface plasmons, one can expect that the number and frequency of the excited modes will reflect the recursive pattern of the metallic layer; overall, a feature-rich surface will result in feature-rich reflection spectrum due to the multiple SPP resonances. Dettmann [27] has used a Fourier transform to describe the Cantor set charge distribution and its accompanying electrostatic potential, which has been shown to be a dimension-dependent power law. Thus, the non-integer fractal dimension of the structure is a quantity that might be preserved to some degree in the reflection spectrum. However, the exact analytical relation between structure and spectrum dimension is nontrivial; every metallic surface supports multiple plasmonic modes, generating many overlapping spectral lines of various strength and width. The proposed system is one of the simplest models of a structure where the dimension can be precisely controlled with parameter *f* and the reflection spectrum is a result of well-known resonances given by (2), providing a convenient model and a basis for our further dimension analysis in systems with more complicated geometry.

## 3. Numerical Results

To calculate the reflection and scattering spectra, we have used the FDTD method, with medium parameters described in [24,26]. The simulation domain is a two-dimensional cross-section of the structure, as shown in Figure 1a. The whole domain is divided by a rectangular grid with a single cell size Δx. At every grid point, the electric and magnetic field distributions $\vec{E}\left(x,y,t\right)$, $\vec{H}\left(x,y,t\right)$ are calculated from their previous values $\vec{E}\left(x,y,t-\Delta t\right)$, $\vec{H}\left(x,y,t-\Delta t\right)$ with evolution equations derived directly from Maxwell’s equations. In the chosen, two-dimensional system, one has three non-zero field components $\vec{E}=\left[E_{x},E_{y},0\right]$ and $\vec{H}=\left[0,0,H_{z}\right]$. The computational domain is a square grid, 1000 × 1000 unit cells. For this geometry, the most efficient use of available space is to place the metallic layer at the diagonal and use 45-degree incidence angle. In such a system, the incident field propagates only along *x* axis and has a single non-zero electric field component $E_{y}$ whereas the reflected beam electric field is $E_{x}$. This allows for easy decoupling of incident and reflected fields for further analysis. The domain is terminated with absorbing layers having a reflection coefficient smaller than $10^{-4}$. The structure is finite and contained within the domain in (x,y) plane. Since the simulation is two-dimensional, the system is assumed to be semi-infinite (much larger than the computational domain) in the *z* direction. The glass is assumed to have a constant susceptibility $\epsilon_{g}=2.25$ while the metal is described by the Drude model (3). We have used ADE (Axillary Differential Equations) approach [28], where we compute medium polarization by solving a second-order PDE in the form

(5) $$\ddot{P}+\gamma \dot{P}=\frac{\omega_{p}^{2}}{\epsilon_{\infty }}E,$$

with the fitted medium parameters: plasma frequency $\omega_{p}$, damping constant γ and high frequency susceptibility limit $\epsilon_{\infty }$. The full set of equations solved in our FDTD approach is as follows

(6) $$\begin{array}{ccc} \frac{\partial E_{y}\left(x,y,t\right)}{\partial x}-\frac{\partial E_{x}\left(x,y,t\right)}{\partial y} & = & -\mu_{0}\frac{\partial H_{z}\left(x,y,t\right)}{\partial t},\\ \frac{\partial H_{z}\left(x,y,t\right)}{\partial x} & = & -j_{y}\left(x,y,t\right)-\epsilon_{0}\frac{\partial E_{y}\left(x,y,t\right)}{\partial t}-\frac{\partial P_{y}\left(x,y,t\right)}{\partial t},\\ \frac{\partial H_{z}\left(x,y,t\right)}{\partial y} & = & j_{x}\left(x,y,t\right)+\epsilon_{0}\frac{\partial E_{x}\left(x,y,t\right)}{\partial t}+\frac{\partial P_{x}\left(x,y,t\right)}{\partial t}, \end{array}$$

where $P_{x},P_{y}$ are components of polarization vector calculated from (5), *j* is the current density, $\epsilon_{0},\mu_{0}$ are the vacuum permittivity and permeability. The above equations are rearranged to obtain time derivatives of the $E_{x}$, $E_{y}$, $H_{z}$ fields, which are then used to calculate the field evolution with some constant time step Δt.

As mentioned before, due to the finite spatial resolution of the simulation Δx limiting the size of the smallest features, the Cantor set structure is generated in 3 iterations.

**Figure 3:**
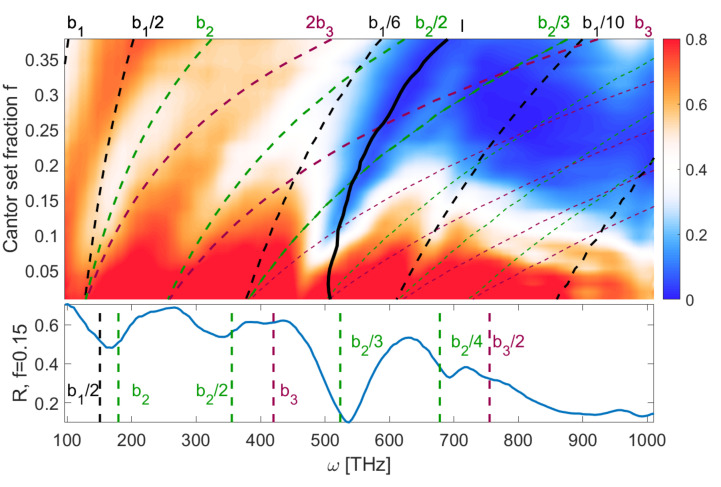
Reflection coefficient as a function of frequency and the Cantor set fraction *f*, for d=0 and h=45 nm. Black, green and magenta lines mark the plasmonic modes corresponding to first, second and third iteration structures.

**Figure 4:**
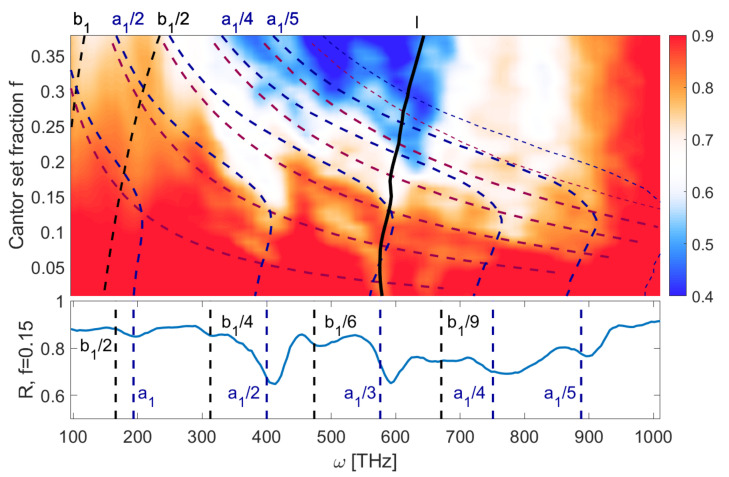
Reflection coefficient as a function of frequency and the Cantor set fraction *f*, for d=45 nm and h=90 nm. Black lines mark the island modes. Blue and magenta lines are the groove modes with and without low *f* correction, respectively.

The Figure 3 shows the reflection spectrum in a system where d=0 and h=45 nm, e.g., the indentations are cut through the whole metal layer, forming islands of metal on the glass substrate. The range of values of *f* describes structures varying from continuous metal layer (f=0) to small islands covering 21.6% of glass surface (f=0.4). One can see multiple minima of reflection (shown in white and blue color), which correspond to the predicted plasmonic modes marked by dashed lines. These frequencies are calculated as follows: for any given length of metallic structure $b_{i}$, there are multiple matching standing-wave modes given by Equation (2). By using the dispersion relation (4), we calculate the mode frequencies $\omega_{n}\left(\lambda_{n}\right)$. The calculated modes are identified in Figure 3 and Figure 4 by their wavelength shown on the top. In Figure 3 one can see that the frequencies are increasing with a fraction *f* due to the fact that the metal islands and corresponding plasmon wavelengths become smaller. As mentioned before, the best fit is obtained when taking into account the sizes of structures in multiple Cantor iterations. Additionally, the spectrum contains a wide minimum (thick, black line, marked *l*) which is a generic plasmonic mode of an uncut metal layer [24]. The large area of low reflection coefficient for ω>500 THz and f>0.1 is a result of many overlapping plasmonic lines in this region; one can see that the local minima of reflection correspond to the crossing points of these lines. Overall, the reflection coefficient is proportional to the surface of metal and thus decreases with *f*. In the limit of f=0, only generic, continuous layer mode and the lowest order island modes $b_{1}$, corresponding to the whole structure size *l*, are present. The close correspondence between reflection minima and predicted SPP resonances is more apparent in the cross-section shown in Figure 3 bottom panel. The two strongest resonances are the generic line overlapping with $\lambda =b_{2}/3$ line and the combination of $b_{2}$ and $b_{1}/2$ lines.

The next analyzed system consists of deep grooves with h=90 nm cut in a thick metal layer, resulting in minimum thickness d=45 nm. In this case, one can expect not only the “island” modes, but also “groove” modes forming inside the indentations. This is shown in Figure 4. The reflection spectrum depends mostly on the groove modes, which are the strongest plasmonic resonances due to the fact that the metal layer is thinnest inside the indents. One could expect that in the limit f→0, the indents and corresponding wavelengths $\lambda_{i}$ become negligibly small, and $\omega_{i}\to \infty$. Such modes are marked by magenta lines. However, as the grooves become narrower, the plasmonic modes start to form on their vertical sides, with constant wavelength Nλ/2=h. We have taken this into account by introducing effective groove size *l* which approaches *h* as f→0. This results in the characteristic shape of the blue lines seen in Figure 4, which are not divergent for f→0. The generic mode, again marked by thick, black line, is relatively weak due to the large effective thickness of the metal layer, especially in the limit of f→0. On the cross-section at the bottom of the figure, one can notice that some reflection minima are blue-shifted as compared to the predicted frequencies; as shown by Novotny [21], there is a correlation between a shape of emitting elements and wavelength, which at optical frequencies may be smaller than the structure length by as much as 20%.

## 4. Calculation of Dimension

**Figure 5:**
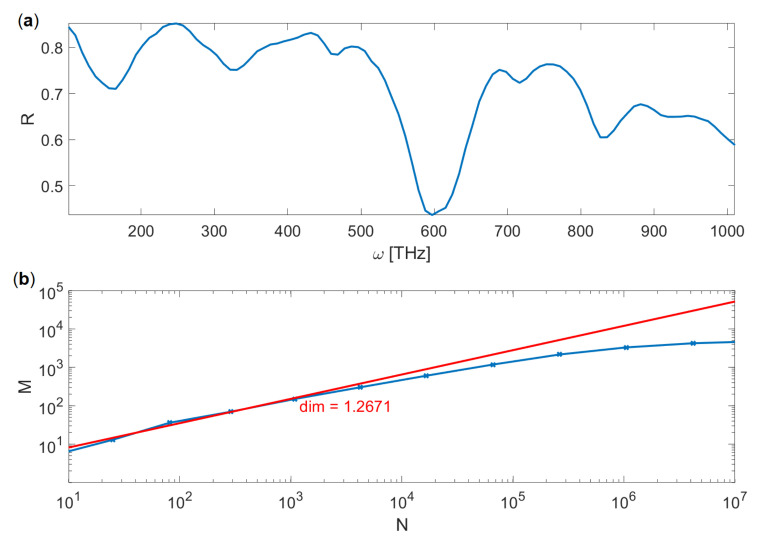
(**a**) Numerically calculated reflection coefficient for d=0, h=45 nm, *f* = 1/3. (**b**) The fractal dimension of the curve determined by fitting (red line), according to the Equation (7).

Our numerical results consists of discrete set of frequencies $\omega_{i},\;i=1\ldots 200$, and the corresponding values of reflection coefficient $R_{i}\left(\omega_{i}\right)$. These pairs can be interpreted as a set of points on a two-dimensional plane, which can be connected to form a piecewise linear curve, as shown in Figure 5a, where reflectance of the first discussed system for *f* = 1/3 is shown. In other words, consecutive data points connected with negligibly thin lines are interpreted as a two-dimensional, geometrical object in [ω,R] coordinate space.

We employ a box-counting method [29,30] to calculate the fractal dimension of this curve. Let’s consider a rectangular region of space enclosing the curve and define a grid that divides this space into rectangles (boxes) of a size ζ. The side of the whole grid is $N∼\zeta^{-1}$ boxes long. Then, we count the number of boxes *M* which are non-empty, e.g., they contain some part of a curve, which can be either a data point $\left[\omega_{i},R_{i}\right]$ or a fragment of a line connecting such points. The so-called Minkowski-Bouligand dimension *D* is determined by observing how the number of non-empty boxes *M* scales with their size ζ. For example, for a simple, one-dimensional structure such as infinitely thin straight line, one would obtain $M∼\zeta^{-1}$ and for two-dimensional area, $M∼\zeta^{-2}$. One can see that the exponent determines the dimension; therefore, we can use a general expression [29]

(7) $$D=\frac{\partial \log M}{\partial \log N}.$$

Numerically, we estimate the dimension *D* by plotting the function logM(logN) and fitting a straight line, as shown in Figure 5b. One can see that in the limit of ζ→0 (e.g., large number of boxes N→∞) the function slope (e.g., dimension) is decreasing. This is caused by our numerical approach, where the curve is represented by a finite number of pixels $M_{max}$; when the box size approaches the pixel size, the *M* stops increasing and the curve effectively becomes a set of separate, zero-dimensional points.

**Figure 6:**
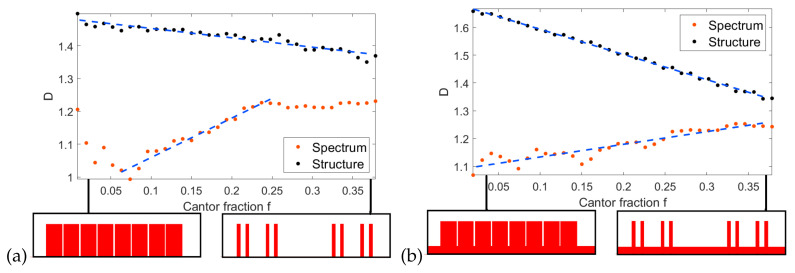
Calculated dimension of the reflection spectrum for (**a**) d=0,h=45 nm and (**b**) d=45 nm, h=90 nm. Insets: schematic of the structure.

The Figure 6a shows the calculated structure and spectrum dimensions *D* as a function of the fraction *f*. The numerical results are marked by points; due to the fact that the exact value of the calculated dimension depends on many factors such as the number of points in the spectrum, numerical accuracy and choice of points in the fitting, straight lines are added to the plots to emphasize the general tendencies. We have found that these tendencies are relatively independent of numerical considerations and are preserved when scaling the system. As expected, the dimension of the structure is decreasing with *f*; one obtains a continuous metal layer at f=0 and a set of separate, point-like islands for large *f*. The dimension of the reflection spectrum also shows a linear dependence on *f*, but only for f<0.25. For a very low *f*, the reflection spectrum is simple, similar to the case of a continuous layer, which results in a low dimension D∼1. For f<0.05, small gaps between islands cannot be adequately resolved on the numerical grid, leading to spurious results. As the gaps increase, the resulting large metal islands produce multiple, strong plasmonic modes, producing rich reflection spectrum which approaches the asymptotic value of D∼1.25 for f≈0.25. Further increase of spacing between islands does not add any new features; as the metal islands become smaller, resonant modes shift to higher frequency and eventually become undetectable. This results in a constant spectrum dimension for f>0.25. Figure 6b shows a very similar tendency. However, in this case, it extends to the whole range of *f* because the spectrum remains relatively feature-rich for all values of *f* due to the interplay between island and groove modes. As in Figure 1a, the results for structures with small *f* show the highest variance which is caused by finite numerical accuracy. Due to the overall low reflection coefficient of these structures, the reflection spectrum cannot be calculated reliably enough. Also, the spatial features of structures with small *f* are comparable in size to the numerical grid, which introduces rounding off errors and aliasing. These factors produce visible, quasi-periodic variations depending on the size of numerical spatial and time steps.

**Figure 7:**
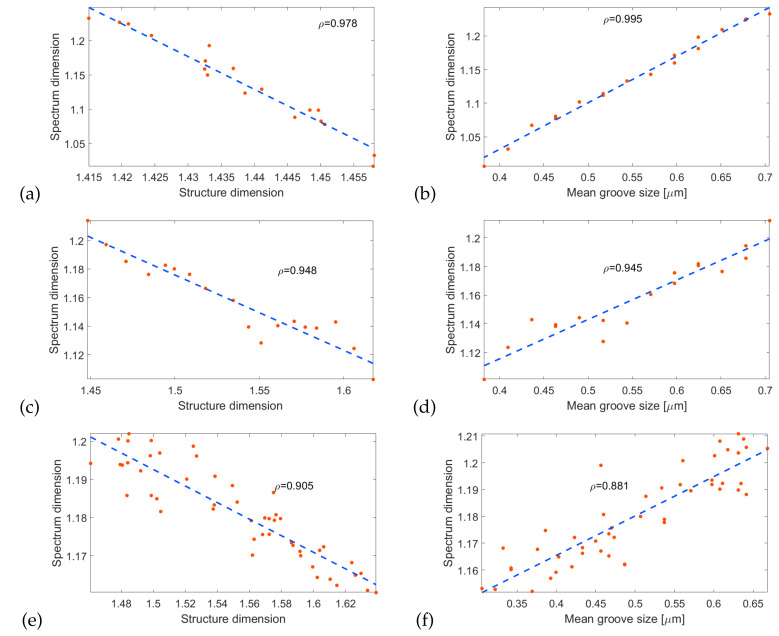
Calculated dimension of the reflection spectrum for (**a**,**b**) d=0,h=45 nm and (**c**–**f**) d=45 nm, h=90 nm, as a function of structure dimension and mean groove size.

Due to the linear dependence of the structure and spectrum dimension on *f*, one can calculate the correlation between these two quantities. One of the most commonly used measures of correlation is the Pearson correlation coefficient $\rho \left(x,y\right)=\frac{cov(x,y)}{\sigma (x)\sigma (y)}$, where cov(x,y) is the covariance of quantities *x*, *y* and σ(x) is the standard deviation of *x* [31]. Figure 7a shows a linear fit to the relation between spectrum and structure dimension in the first considered system (d=0,h=45 nm), with ρ=0.978 indicating that there is a strong correlation between these variables. Moreover, a very good fit is obtained for the spectrum dimension and mean groove size (Figure 7b). This result extends also to the island size and effective layer thickness, as these geometrical features are all directly linked—the sum of all islands and grooves is the structure length *l* and the thickness is a weighted mean of *d* and d+h, where the weight is the ratio of the total island surface to total groove surface. In the discussed case of d=0, the islands are separate metallic structures on glass substrate and the mean groove size is the mean distance between these structures. This means that the results could be applicable to random ensembles of nanoantennas [32,33,34] and other similar systems [35]. Figure 7c,d depict the same fits performed for the second system (d=45 nm, h=90 nm). The straight lines have the same direction as in the previous case, which indicates that the results are general and repeatable across various systems, despite different absolute values of the structural dimension and spectrum dimension. Finally, we have performed simulation for semi-random structure, where the island and groove sizes are random variables with expected values given by Equation (1) and a standard deviation of 30%. The islands have a height h=90 nm and the thickness of the continuous layer is d=45 nm. Such a system represents a realistic, rough metallic surface. Figure 7e,f shows the correlations obtained in this setup. Due to the complicated geometry with many different distances in the structure, there are overall more plasmonic modes than in previous systems. As a result, the reflection spectrum is richer and its dimension is larger. There is also less variance in the value of dimension—even for small *f*, the structure can support a large number of plasmonic resonances, so that the spectrum does not become significantly simpler for f→0.

To sum up, in both discussed cases the reflection spectra contain a large number of plasmonic modes which can be associated with specific geometrical features of the metallic structure. Due to this dependence, one can correlate the dimension of the structure and the spectrum. This is the key result of this work. The presented relations are repeatable across different systems and hold in the presence of noise. These results suggest that the proposed approach is viable for realistic, random and semi-random fractal-like structures. This facilitates the use of fractal dimension analysis as a useful tool to extract information about the structure from the spectrum, even for the case of a large number of overlapping plasmonic modes. Such correlations could be useful in surface physics [35] because the spectroscopic interpretation of the spectrum enables one to get insight into the material structure of the illuminated system. Moreover, we have shown that the frequencies of plasmonic modes depend on *f*(Figure 3 and Figure 4), which in turn affects the structure dimension in a linear manner (Figure 6). Therefore, one can associate the dimension with frequency shifts, as reported in [30].

It should be mentioned that apart from *f*, another degree of freedom in structure generation is the number of Cantor set iterations. We have shown that particular plasmonic modes can be associated with iteration number and therefore the total number of modes is directly dependent on the maximum number of iterations. This is a known effect in superlattices [19]. In studies of light scattering on Cantor set, the intensity spectrum follows iteration-dependent power laws [14].

## 5. Correlation Between Dimension and Structure Entropy

It has been shown that the Shannon entropy of a structure is closely related to its fractal dimension [15]. In the case of our Cantor set, the structure is numerically represented by a vector of discrete values indicating the thickness of the metal layer along the surface. Specifically, in FDTD calculations we have used *N* = 300 values $d_{i},i=1\ldots N$, which are either equal to $d_{i}=d$ or $d_{i}=d+h$. Therefore, one can conclude that the surface is encoded by *N* bits of information. To calculate entropy, we use the standard formula [36]

(8) $$S=-\sum_{i=1}^{N}p_{i}\log_{2}p_{i},$$

where $p_{i}$ is the probability of $d_{i}$ having the given value; for Cantor fraction *f*, one has

(9) $$p_{i}=\left\{\begin{array}{cc} f, & d_{i}=d,\\ 1-f, & d_{i}=d+h, \end{array}\right.$$

which results in the total entropy

(10) $$S=-N\left[f^{2}\log_{2}f+{\left(1-f\right)}^{2}\log_{2}\left(1-f\right)\right].$$

**Figure 8:**
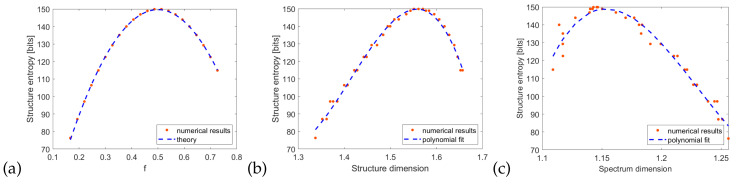
Structure entropy (8) as a function of (**a**) fraction *f* (**b**) structure dimension (**c**) spectrum dimension.

The relation above is a function close to a parabola, with a maximum value of N/2 at f=0.5. The comparison of *S* calculated from Equation (8) and a theoretical relation (10), obtained for the second structure, e.g., d=45 nm, h=90 nm, is shown on the Figure 8a. The results indicate that the system exhibits varying degrees of order, especially in the case of very low or very high *f* the structure collapses to the trivial case of smooth surface. The peak entropy is half of its maximum possible value, e.g., S=N, which would be obtained in a perfectly random structure. As shown before, there is a linear dependence between Cantor fraction *f* and the structure dimension. Therefore, the entropy as a function of dimension shown in Figure 8b has a similar shape to Figure 8b. Interestingly, the best fit is obtained with a third-degree polynomial and the peak entropy occurs for D=1.56, which is roughly halfway between closest integer dimensions D=1 and D=2. One can conclude that the fractal structure is the richest, in terms of Shannon’s information, when the dimension is possibly far from the integer values. Finally, one can express the structure entropy as a function of its reflection spectrum dimension (Figure 8c). Again, a third-degree polynomial with a maximum value at D=1.15 is the best fit and the quality of the fit is better for the more complicated spectra characterized with a larger dimension.

**Figure 9:**
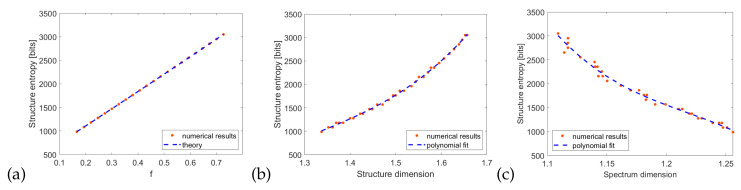
Structure entropy (11) as a function of (**a**) fraction *f* (**b**) structure dimension (**c**) spectrum dimension.

The entropy calculated with Equation (8) is based on a one-dimensional map of height values, which is a minimal information needed to describe the metallic layer. However, one can also use the direct representation of the structure in the FDTD simulation, e.g., a two-dimensional binary map describing the area with and without metal. In such a case, we have

(11) $$S=-\sum_{i=1}^{N}\sum_{i=1}^{M}p_{i}\log_{2}p_{i},$$

where the area has a size N×M. In such a case, the dependence of entropy on the fraction *f* becomes linear (Figure 9a). The relation between entropy and dimensions (Figure 9b,c) is also very close to a linear function, which has been proposed in [15], in a more general context of Rényi entropy and generalized fractal dimension. The deviation from the straight line is caused by finite accuracy of box-counting dimension calculation, specifically the choice of points to fit the asymptote shown on Figure 5. This is caused by the fact that the entropy value of a fractal system depends on the scale of measurement (in our case, number of points *N*), but the fractal dimension is independent of the scales [16]. Finally, from Figure 9c we conclude that it is possible to estimate the structure entropy not only from its fractal dimension but also from the dimension of its reflection spectrum.

It should be stressed that our results indicate that the structure entropy, which is based on the spatial distribution of metallic elements, manifests itself in the reflection spectrum, which is a Fourier transform of the time-dependent signal. This contrasts with a more direct approach such as [17], where the authors have studied the relation between structure entropy and the spatial distribution of reflected wave.

## 6. Conclusions

We have shown that a fractal plasmonic system in the form of Cantor set supports multiple standing wave SPP modes which are closely linked to the specific geometrical features of the structure. This dependence allows one to predict the location of extrema in the reflection spectrum. Moreover, it is demonstrated that by interpreting the reflection spectrum as a fractal, one can calculate its non-integer dimension and relate it to the dimension of the illuminated structure. This provides a novel way to extract information about structure from the reflectance. Importantly, the proposed approach based on the spectrum dimension analysis provides clear correlations even in the case where individual resonances cannot be easily resolved. The results could be applied to other systems with non-integer dimensions, including rough-surfaced metallic layers. The presented scheme is based on the well-known Kretschmann configuration and the simple, single-layer system geometry seems to be within reach of the usual fabrication techniques. Experimental data for the discussed problems are not available yet but we hope that our theoretical results will pave the way for future observations and might have practical applications in, e.g., construction of photodetectors, surface-enhanced spectroscopy, photovoltaic devices, and high-gain, compact, multiband antennas [9].
